# Supplementary material for: Solubility Enhancement of Atrazine by Complexation with Cyclosophoraose Isolated from Rhizobium leguminosarum biovar trifolii TA-1
Source: Polymers (Basel). 2019 Mar 12;11(3):474. doi: 10.3390/polym11030474 (PMC6473739; doi:10.3390/polym11030474)
Supplement: Supplementary file 1 [file polymers-11-00474-s001.pdf]

Supplementary Materials

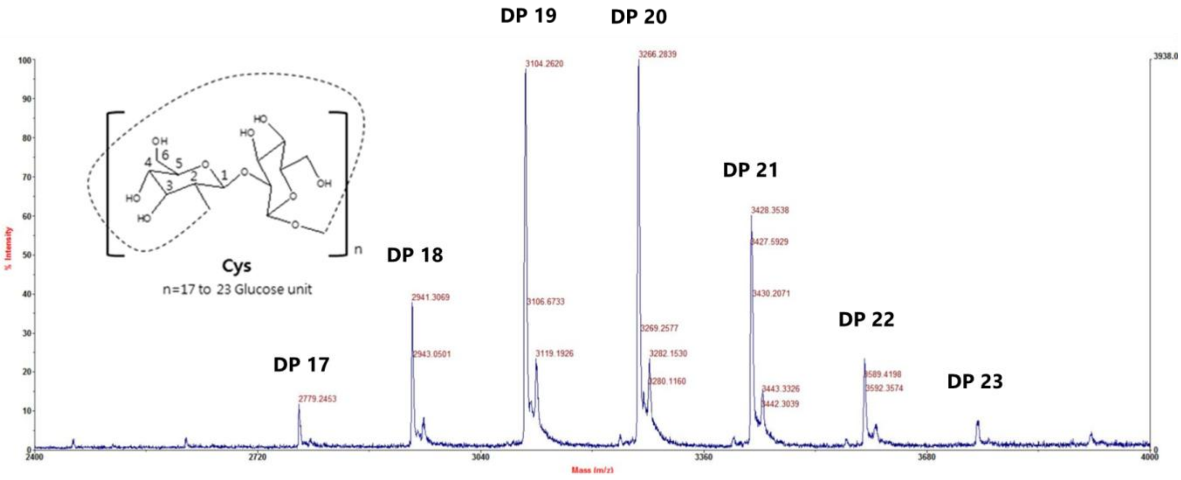

Figure S1. MALDI-TOF mass spectra of Cys.

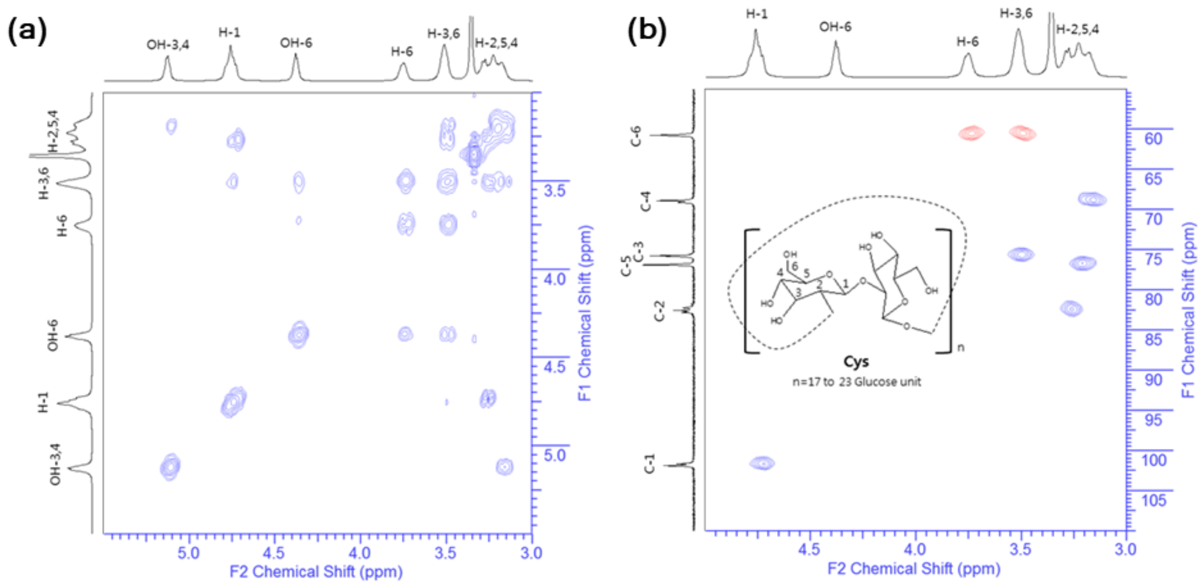

Figure S2. NMR spectroscopic analyses of Cys. $^1H-^1H$ correlation spectroscopy (COSY) (a), $^1H-^{13}C$ heteronuclear single quantum coherence (HSQC) (b).

The COSY also confirmed the clear correlation between the each protons of Cys (Figure S1a). In addition, the HSQC spectrum (Figure S1b) shows that the C-6 carbon (61.2 ppm) was correlated with H-6 (3.75 ppm) and H-3,6 (3.51 ppm) protons, respectively, and the C-3 carbon (76.8 ppm) was correlated with H-3,6 (3.51 ppm) protons. Also the spectrum shows crosslinking between three peaks of H-2,5,4 and carbons (C-2, C-5, and C-4) in order. The structural analyses support the successful production of Cys from the *R. trifolii* TA-1.

Table S1. Chemical shift (ppm) of the protons of atrazine and Cys in the free and inclusion complex state.

proton	$\delta/10^{-6}$		
	δ_1[a]	δ_2[b]	$\Delta\ \delta$[c]
Atrazine			
a-NH	7.7795	7.7705	-0.009
b-NH	7.7205	7.711	-0.0095
c-H	4.007	4.002	-0.005
d-H	3.230	3.225	-0.005
e-H	1.113	1.1085	-0.0045
f-H	1.0735	1.069	-0.0045
Cyclosophoraose			
OH-3,4	5.123	5.117	-0.006
H-1	4.759	4.7555	-0.0035
OH-6	4.380	4.3715	-0.0085
H-6	3.753	3.751	-0.002
H-3,6	3.5135	3.5105	-0.003
H-2	3.2745	3.2718	-0.0027
H-5	3.229	3.2245	-0.0045
H-4	3.1795	3.176	-0.0035

[a]Free state, [b]Inclusion complex, [c]$\Delta\ \delta=\delta_2-\delta_1$.

^{1}H NMR experiment was performed to measure chemical shifts of atrazine by Cys upon the inclusion complexation. In the case of inclusion complex, whole protons are shifted into upfield direction. Not only a-NH proton of atrazine, but also b-NH proton is clearly shifted from 7.7205 to 7.711 ppm. In addition, H-5 proton, which correlated with b-NH of atrazine proton in the ROESY experiments, is shifted from 3.229 to 3.2245. Chemical shift changes of Cys and atrazine protons were summarized in Table S1. The chemical shifts indicate that the protons of atrazine were surrounded by an electron density of Cys upon the inclusion complexation. This phenomenon has often been observed in typical inclusion complexation study [10, 43]. Among the protons of atrazine, the b-NH was more affected by Cys than other protons. However, Cys showed a structurally different binding mode toward atrazine in aqueous state. Specifically, Cys showed more favorable accessibility for the a-NH (ethyloamino proton) of atrazine than other moieties upon the complexation.

Table S2. Calculated data of phase solubility for K_c of Cys, α-CD, β-CD, and γ-CD.

	S_0(M)	slope	K_c (M^{-1})
Atrazine/Cys	2.48	0.33	206.56
Atrazine/α-CD	2.64	0.11	50.17
Atrazine/β-Cys	2.64	0.10	44.18
Atrazine/γ-Cys	2.53	0.10	46.12

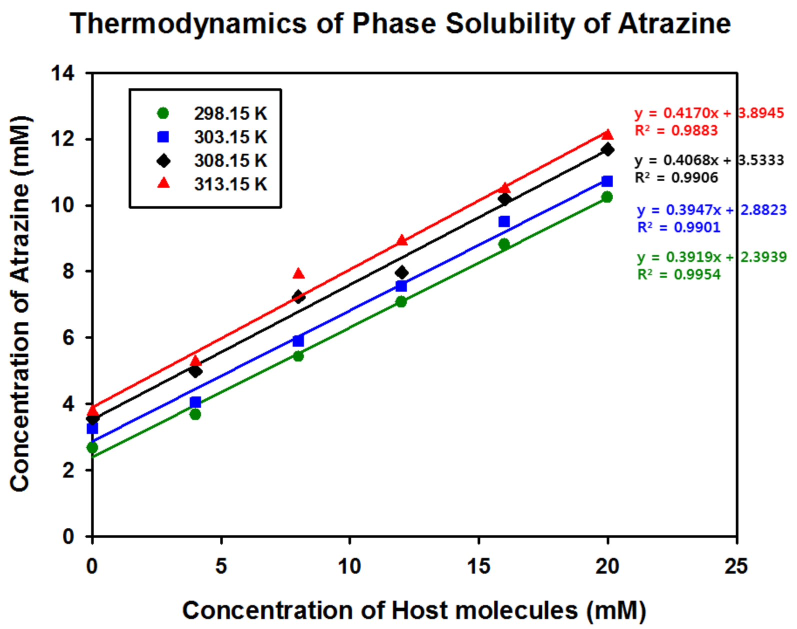

Figure S3. Phase solubility diagram of atrazine with Cys at 298.15, 303.15, 308.15 and 313.15 K.

Table S3. Calculated data of phase solubility for K_c, ΔG^0, ΔH^0, and ΔS^0 at 298.15, 303.15, 308.15 and 313.15 K.

	T (K)	S_0(M)	slope	K_c (M^{-1})	ΔG^0(kJ/mol)	ΔH^0(kJ/mol)	ΔS^0(kJ/mol*K)
Atrazine/Cys	313.15	3.75	0.41	190.38	-13.66	-2.00	0.03
	308.15	3.55	0.40	192.77	-13.48		
	303.15	3.26	0.39	199.60	-13.34		
	298.15	2.67	0.39	241.26	-13.59		
